# The Fate of Fusions

**DOI:** 10.3390/cells8010013

**Published:** 2018-12-29

**Authors:** Gary Clawson

**Affiliations:** Hershey Medical Center, Pennsylvania State University, Hershey, PA 17033, USA; gac4gac4@gmail.com; Tel.: +1-717-531-5632

**Keywords:** macrophage-tumor cell fusions, MTFs, CTCs, metastasis, metastasis-initiating cells, MICs

## Abstract

The concept of leukocyte-tumor cell fusion as a significant driver of cancer progression has been around a long time, and has garnered growing support over the last several years. The underlying idea seems quite simple and attractive: Fusion of tumor cells (with their inherent genetic instability) with leukocytes, particularly macrophages, could produce hybrids with high invasive capabilities, greatly facilitating their metastatic dissemination, while potentially accelerating tumor cell heterogeneity. While there are a number of attractive features with this story on the surface, the various studies seem to leave us with a conundrum, namely, what is the fate of such fusions?

## Introduction and Discussion

The concept of leukocyte-tumor cell fusion as a significant driver of cancer progression has been around a long time, and has garnered growing support over the last several years, with numerous reports describing what appear to be hybrid cells. There are a variety of potential mechanisms by which such hybrid cells could arise. However, while other processes such as trans-differentiation, and developmental mimicry, etc. could give rise to similar apparent hybrid cells, these processes provide no explanation for the highly abnormal ploidy of the hybrid cells, leaving fusion as the most likely explanation [1]. There are a few specific examples in human cancers in which fusion has been demonstrated convincingly [2,3,4], which were documented in bone marrow transplant recipients. The underlying idea seems quite simple and attractive: Fusion of tumor cells (with their inherent genetic instability) with leukocytes, particularly macrophages, could produce hybrids with high invasive capabilities, greatly facilitating their metastatic dissemination, while potentially accelerating tumor cell heterogeneity. While there are a number of attractive features with this story on the surface, at present there is little actual documented support for it, and in fact it creates a conundrum. In the end, we wind up wondering what the fate of fusion is?

There are a considerable number of reports over the years describing leukocyte-tumor cell fusions (LTFs), most often macrophage-tumor cell fusions (MTFs), in human cancers [2,4,5,6,7,8,9,10,11]. In general, the mechanism(s) by which the apparent hybrid cells arose were not clearly delineated for various reasons.

In a recent study [12], Gast et al. clearly documented a fusion mechanism for production of MTFs, both in vitro and in a mouse model. Gast et al. [12] went on to identify apparently analogous MTFs in human cancers, particularly pancreatic ductal adenocarcinoma (PDAC), and in addition, using a small cohort of PDAC patients, they found that circulating high levels of MTFs were associated with patient stage, and with a statistically significant decrease in survival time [12].

Perhaps the most surprising aspect of MTFs is their sheer prevalence. In our initial report, we used a microfabricated filter device to capture circulating tumor cells (CTCs) [13]. With melanoma CTCs, approximately 50% of captured CTCs showed dual-staining for leukocyte-tumor cell markers (i.e., apparent MTFs). Further work showed that these MTFs appeared to be macrophages both morphologically and ultrastructurally, yet they showed highly abnormal DNA contents and contained melanoma-specific mutations in the B-Raf gene in patients whose melanomas contained such mutations [10]. However, nearly all CTCs captured from PDAC (and colorectal cancer) patients dual-stained for macrophage and tumor cell markers [11], quite consistent with the 90% hybrid cells in PDAC patients reported by Gast et al. [12]. In addition, we consistently found that large subpopulations of cells appear to be MTFs in human melanomas ([10]; both primary and metastatic lesions) and PDACs [11], analogous to findings by Gast et al. [12] in primary PDACs.

However, these findings actually raise a number of concerns regarding the role of MTFs in cancer progression, and particularly metastasis, which really need to be addressed. Metastasis is generally thought to occur as a “cascade” with sequential steps. Phase 1 basically encompasses the physical journey of the CTC to distant tissues, while phase 2 (which is even more complicated and less well understood) encompasses adaptation of translocated CTCs to their new environments, with subsequent proliferation to produce a typical metastatic cancer lesion [14,15,16]. While on the surface, production of leukocyte-tumor cell hybrids seems to provide a convenient explanation for metastasis, the assumption that such hybrids are responsible for production of metastatic lesions in turn seems to lead to inconsistencies. Firstly, metastasis is an inherently inefficient process [17,18,19,20,21,22], but one would assume that these motile, highly invasive MTFs would be very effective at producing metastatic foci, at least at the phase I stage This is compounded by the fact that circulating MTFs in PDAC and other cancers seem to be an order of magnitude more plentiful than “conventional” CTCs, as determined by CellSearch. Considering “conventional” CTCs for example, if there are 5 CTCs/7.5 ml of a patient’s blood, a simple calculation using a CTC circulation half-life of 1–2 h [23] would imply that 40–80,000 conventional CTCs circulate every day (manuscript in preparation), yet very few if any metastatic foci (even phase 1) develop from them. However, with MTFs approximately an order of magnitude higher than conventional CTCs, that would mean something like 500,000 MTFs are unsuccessful at accomplishing even a “phase 1” landing on any given day, even given their high motility and invasive capabilities. This doesn’t seem to make any inherent sense. There is also another issue regarding CellSearch: CellSearch has established prognostic value clinically in a variety of cancers, in spite of variety of recognized shortcomings (like loss of the epithelial marker EpCam expression on CTCs after the epithelial-to-mesenchymal transition, etc.), and even problems with definition of CTCs [24], and conventional CTC measurement seems to have clinical value even in PDAC [25,26,27,28,29]. However, CellSearch eliminates MTFs by definition, thus, is there just an accidental stochastic relationship between MTFs and conventional CTCs to explain this? In general, the detection of CTCs in PDAC patients is surprisingly low in many cases [26,29], perhaps because nearly all CTCs are actually MTFs.

Another issue would seem to be the prevalence of MTFs in primary vs. metastatic lesions. Although the literature on this matter is sparse at best, metastatic lesions do not seem to be enriched for MTFs vs. primary tumors. In human melanoma samples, for example, there wasn’t any notable difference in the proportion of MTFs in primary melanomas vs. their metastases (it was surprisingly high in both), although this is anecdotal and no quantitation was done [10].

There are a number of experimental reports indicating that tumor cell fusions may promote more aggressive behavior [30,31,32,33], although this is not universal [34]. We have performed transplantation experiments in nude mice using MTFs cultured from the blood of melanoma and PDAC patients. Melanoma MTFs were transplanted subcutaneously, and after a few weeks no tumor was found at the inoculation site. We observed occasional MTFs in adjacent subcutaneous tissue (although not at the injection site) and in various stromal locations, as well as foci of cells growing in the pancreas [10]. However, the pancreatic islands of cells were very odd, not recapitulating melanomas at all, but rather consisting of what appeared to be well circumscribed collections of well-differentiated cells (sometimes pigmented) which expressed a number of human-specific markers, including melanocytic markers (MLANA, ALCAM) and M2-macrophage markers (CD206, CD208). Transplantation experiments with MTFs cultured from PDAC patient blood [11] produced similar results, in that no tumor was found at the orthotopic injection site (which was intra-pancreatic). Mice also showed analogous well-differentiated islands of cells in the pancreas which stained for human pancreatic, stem cell, and M2-macrophage markers (since the MTFs were transplanted orthotopically into the pancreas in these experiments, it cannot be concluded that they were “metastatic”). However, the pancreatic foci did not grow or progress over time (4–12 weeks). The only other MTFs found in the mice were single cells or clusters of a few cells found focally in the liver, spleen, lungs, and subcutaneous tissues. There was no development of metastatic lesions over time. Thus, although they clearly disseminated, MTFs did not form overtly cancerous metastatic lesions in the traditional sense, and no primary tumors developed at the transplantation sites.

There are a couple of caveats which could complicate these experiments. For one, the MTFs we transplanted had been grown in culture for 4 weeks or so. The culture conditions could have affected their subsequent behavior in vivo. However, to this point, others have found that even large numbers of CTCs harvested by leukapheresis, which presumably contained substantial numbers of MTFs, were surprisingly unable to form metastatic foci [35].

The other point which certainly merits consideration is the use of immune-compromised mouse models. The immune system should be expected to have a major impact on formation of metastatic foci, yet it was lacking in this context. In an “immune-competent” context, the nature of the MTFs is undoubtedly important; in particular, it is noted that the M2 polarization observed in MTFs is generally considered to have anti-inflammatory (immune suppressive) effects, and many recent studies have documented such effects (for example, [36] and see [37]).

These considerations, therefore, seem to leave us in a conundrum. Large numbers of highly motile, highly invasive MTFs in primary tumors and blood would seem to be extremely inefficient at producing metastatic lesions, even phase 1 foci of individual cells, and they were unable to accomplish phase 2 progression of any lesions.

The nature of metastasis-initiating cells (MICs) is not clear, but many would argue that MICs are likely to be derived from cancer stem cells. The MICs could be cancer stem cells, or perhaps represent fusions of prototypical cancer stem cells with macrophages, which would thus represent a very small subset of the surprisingly prevalent overall MTF populations in various tumors. There are certainly many reports which describe development of more aggressive phenotypes upon LTF fusions [30,31,38], although it seems that the properties of the LTFs may be determined very soon after fusion, probably reflecting clonal “soil” selection rather than a continuing phenotypic plasticity [39]. Results with tumor-stem cell fusions have been mixed: With hepatocellular carcinoma cells, fusion with human embryonic stem cells produced tumor-initiating cells [40], whereas fusion of hematopoietic stem cells with esophageal carcinoma cells did not [34], and potential LTFs were not tumorigenic in mouse models even at very high inoculation numbers [35].

It still seems reasonable to propose that MTFs may not actually produce progressive metastatic lesions per se but rather may actually produce an expanded number of phase 1 “niches” suitable for subsequent colonization by MICs [41], whatever they actually are. The most likely mechanism would seem to be secretion of cytokines and chemokines to alter their microenvironment. In particular, with pancreatic ductal adenocarcinoma, macrophage migration inhibitory factor appears to be a prominent component found in primary and metastatic tumors, and it has important functional interactions with CXCR4 (which is a non-cognate receptor) and CD44 stem cell markers [11].
